# Capsule Retentions and Incomplete Capsule Endoscopy Examinations: An Analysis of 2300 Examinations

**DOI:** 10.1155/2012/518718

**Published:** 2011-09-29

**Authors:** Charlotte M. Höög, Lars-Åke Bark, Juan Arkani, Jacob Gorsetman, Olle Broström, Urban Sjöqvist

**Affiliations:** ^1^Department of Medicine, Karolinska Institute, Stockholm Söder Hospital, 118 83 Stockholm, Sweden; ^2^Department of Gastroenterology and Hepatology, Karolinska University Hospital, 141 86 Stockholm, Sweden; ^3^Department of Gastroenterology, Ersta Hospital, 116 91 Stockholm, Sweden; ^4^Department of Medicine, Danderyd Hospital, 182 88 Stockholm, Sweden

## Abstract

*Aim*. To evaluate capsule endoscopy in terms of incomplete examinations and capsule retentions and to find risk factors for these events. *Material and Methods*. This retrospective and consecutive study includes data from 2300 capsule enteroscopy examinations, performed at four different hospitals in Stockholm, Sweden from 2003 to 2009. *Results*. The frequency of incomplete examinations was 20%. Older age, male gender, suspected, and known Crohn's disease were risk factors for an incomplete examination. The PillCam capsule had the highest rate of completed examinations. Capsule retention occurred in 1.3% (*n* = 31). Risk factors for capsule retention were known Crohn's disease and suspected tumor. Complications of capsule retention were acute obstructive symptoms in six patients and one death related to complications after acute surgical capsule retrieval. *Conclusion*: Capsule endoscopy is considered a safe procedure, although obstructive symptoms and serious complications due to capsule retention can be found in a large series of patients.

## 1. Introduction

Capsule endoscopy (CE) has facilitated and improved visualization of the small bowel mucosa. Since its first introduction by Iddan et al. [1], the method has been routinely used primarily in cases of obscure gastrointestinal bleeding (OGIB) and in the evaluation of patients with known or suspected Crohn's disease (CD). Several studies have shown the superiority of detecting small bowel lesions with CE compared to other diagnostic modalities with these indications [2–6].

To perform a high quality examination and maximize the diagnostic yield, the entire small bowel needs to be visualized [7, 8]. Only an average of 83.5% of the examinations are completed, with completion defined as the capsule reaching the caecum during the recording time [9]. This indicates a limitation of the method since the distal part of the intestine remains unexamined in some patients. Prokinetic drugs, as well as purgatives, have been evaluated to improve the completion rate but were shown to be ineffective [8, 10, 11]. There is currently no validated scale to evaluate bowel cleanliness and no common guideline for bowel preparation although poor views, especially in the distal part of the small intestine, are considered a limitation [8, 10, 12, 13].

Capsule retention is the most feared complication of CE [14] and is defined as the presence of the capsule endoscope in the digestive tract for a minimum of 2 weeks or more, or when the capsule is retained indefinitely in the small bowel unless a targeted medical or surgical intervention is initiated. Several studies have recently addressed capsule retention [9, 12, 15–17]. It is important to differentiate capsule retention from incomplete examination, meaning that the capsule did not reach the caecum during the recording time and delayed transit, which is when the capsule is harboured in the same part of the intestine for more than 2 hours [14], which might be difficult to identify in the small intestine. In the general population, the risk of capsule retention ranges from 1.4% to 2.5% [15, 16] and in patients with known CD a 13% risk has been observed [17]. The most common reasons for capsule retention are CD, obstructive tumor, and diaphragm disease due to the side effects of nonsteroid anti-inflammatory drugs [9, 15, 17, 18]. Patients with OGIB are considered to be at the lowest risk for capsule retention, and patients with a history of obstructive symptoms are at the highest risk [14, 17]. Radiological methods, though improving over time, cannot rule out the possibility of an intestinal stricture [19, 20]. A method for detecting strictures by means of a soluble test capsule has been developed (Agile Patency Capsule, Given Imaging), and results so far have been promising [21]. CE is considered a safe procedure, and, even in cases of capsule retentions, obstructive symptoms due to impaction of the capsule or serious adverse advents following surgical removal of the capsule are rare [7, 9, 13, 15].

This retrospective study included all patients undergoing CE in Stockholm and Gotland Counties in Sweden between 2003 and 2009. The primary aim of the study was to evaluate CE in terms of incomplete examinations and capsule retentions in a large unselected population by including all CE performed during these years. Our secondary aim was to characterize the clinical outcomes of patients with capsule retention.

## 2. Material and Methods

This study comprised 2300 small-bowel CE examinations performed at 4 different hospitals in Stockholm, Sweden between June 2003 and December 2009. All CE studies performed in Stockholm County, consisting of 2 million inhabitants, during this time period were included. 

The introduction of CE started at Stockholm Söder Hospital (Center 1) in June 2003. At this hospital, the Given PillCam SB capsule endoscope (Given Imaging, Yokneam, Israel) was used, and 1473 (77%) of the investigations were carried out using this system. Karolinska University Hospital (Center 2) started CE examinations 4 years later (June 2007) and used the Olympus capsule endoscope (Olympus, Tokyo, Japan). At this hospital, 490 (23%) of the examinations were performed. Ersta Hospital (Center 3) also started in June 2007, and at this hospital 302 CE examinations were performed using the Given PillCam SB system. Danderyd Hospital (Center 4) started CE examinations in August 2009, using the MiroCam capsule device (Intromedic, Seoul, Korea) and performed 35 examinations by the end of 2009. 

All centers contributed data on the total number of CE examinations that were performed as well as data on age, gender, indications, view, and findings in patients with incomplete examinations. Centers 1 and 2 (*n* = 1963, 85% of the all CE examinations) also contributed these facts for all patients receiving CE during the study period.

Indications for CE were OGIB, known or suspected CD, suspected tumor and others. Tumor, was suspected when a previous radiological examination, mainly computer tomography, had raised the suspicion or in cases with a clinical suggestion of malignancy, such as weight loss, unexplained fever, laboratory findings, and symptoms pointing towards malignancy. “Other” indication included diarrhea, coeliac disease, and abdominal pain. 

All medical charts and records of the patients referred for CE were reviewed by a gastroenterologist. If the indication for the CE was bleeding, all patients must have had at least one negative upper endoscopy and one normal colonoscopy at a concurrent time. In cases of suspected or known CD, contraindications such as symptoms of small bowel obstruction or known strictures were ruled out. Radiological exclusion of strictures by means of enteroclysis, MRI, or CT was not required before CE. 

Patient preparation included a liquid diet the day prior to the CE examination and nothing by mouth from midnight. After swallowing the capsule, the patients were allowed liquid food after 2 h and an ordinary diet after 4 h. No laxative was given. In a few patients in which slow gastric transit was highly likely (diabetics, inward-patients, patients on opioids, and patients with a previous CE where the capsule remained in the stomach during the whole recording time) a real time viewer was used 1 h after ingesting the capsule, and in the case of visualized gastric mucosa, gastroscopy was performed, and the capsule was manually placed in the duodenum using a Roth-net.

In those patients where the capsule did not reach the cecum and excretion was not witnessed, an abdominal X-ray was obtained about 2 weeks later. Especially during the first years of use, this routine was not always followed, and in those cases patients were classified as “lost” in this study. If the patient witnessed spontaneous passage, the X-ray was cancelled. 

Capsule retention was defined as the capsule visualized inside the small intestine by radiological examinations two weeks after CE or found during abdominal surgery in an obstructed part of the small intestine. Patients with a confirmed, indefinitely retained capsule were mostly referred for surgery and in all cases were closely followed up. Before surgery was initiated, a new radiological control of the retained capsule was usually done, and in a few cases the capsule had passed spontaneously, even after several months of retention, and these patients were excluded from the capsule retention group. The remaining patients with capsule retention are presented in detail in this study. The CE studies were read by gastroenterologists and, to keep the method in fewer hands, no more than two gastroenterologists at each center were involved.

To identify risk factors for capsule retention and incomplete CE examination, a multivariable analysis using logistic regression was made. In this analysis, only patients from Centers 1 and 2 were included since the data of age, gender, and indications for CE were not known for patients with complete CEs at Centers 3 and 4. In this analysis, we also distinguished patients with previously known CD from patients with a suspicion of CD.

To find out if there were any differences between the two most used capsule systems, Olympus and PillCam, we compared the completeness of examinations between Center 1 (using only PillCam) and Center 2 (using only Olympus capsule). Only patients with OGIB as an indication for CE were compared in order to avoid interference of different management between the two centers involved. Also OGIB is the indication least likely to give rise to capsule retention [14]. At both centers, CE is the next examination done after negative gastroscopy and colonoscopy of good quality in the case of OGIB. Both centers used the same preparation and scheme, as described above, during the examination day. Pearson's chi-square tests were used for calculating the significance.

Statistics were calculated using PASW Statistics 18 (IBM Corporation, Somers, USA). The level of significance was set at 0.05, two sided, for all analyses.

This study was approved by the Regional Ethical Review Boards in Stockholm (no. 2010/1819-31/3).

## 3. Results

A total of 2300 CE examinations were performed in Stockholm County between June 2003 and December 2009. CE failed because of technical failures in 6 patients (all PillCam SB), and these patients were excluded. 5 patients were unable to swallow the capsule and declined endoscopic placement of the capsule into the stomach. The overall indications for CE (known for all patients from Centers 1 and 2, *n* = 1957, 85% of all CE examinations) were as follows: bleeding source (*n* = 1034, 53%), suspected CD (*n* = 577, 29%), known CD (*n* = 152, 8%), tumor (*n* = 118, 6%), and others (*n* = 78, 4%). The mean age was 51 years (range 2–99 years) and 57% were female (*n* = 1117). The diagnostic yield was 55%.

### 3.1. Incomplete Examinations

A total of 463 (20%) examinations were incomplete, meaning that there was failure of the capsule to reach the caecum during the recording time (8–11 h depending on the capsule system). The mean age of patients with incomplete examinations was 53 years (range 6–99 years), and 53% were female. Indications for CE were OGIB (*n* = 208, 45%), known or suspected CD (*n* = 191, 41 %), tumor (*n* = 46, 10%), and others (*n* = 18, 4%). The most common finding was CD (*n* = 104, 22% of examinations). Other findings were slow gastric transit where the capsule remained in the stomach for the whole recording time (*n* = 63, 14%), vascular disease (*n* = 37, 8%), tumor (*n* = 20, 4%), and others (*n* = 74, 17%). 164 (35%) examinations were normal. 77 (17%) patients were lost for followup after incomplete CEs. The diagnostic yield was 51%.

### 3.2. Risk Factors for Incomplete Examination

The risk of incomplete examination (analysed for patients from Centers 1 and 2) was higher for male patients with an odds ratio of 1.34 (1.08–1.67, *P* = 0.009) and increased with age with an odds ratio of 1.02 per year (1.01–1.02, *P* < 0.001). The odds ratio for an incomplete examination was significantly elevated for patients with both suspected and known CD, suspected tumor, and other indication compared to OGIB (Table 1).

### 3.3. Capsule Retentions

Capsule retention was found in 31 (1.3%) patients (Table 2). The mean age was 51 years, and 47% were female. Indications for CE in this group were OGIB (*n* = 10, 32%), known or suspected CD (*n* = 16, 52%), tumor (*n* = 4, 13%), and others (*n* = 1, 3%). Findings were CD (*n* = 15, 48%), tumor (*n* = 6, 19%), stricture (non-CD; *n* = 4, 13%), erosions (*n* = 2, 6%), on-going bleeding (*n* = 1, 3%), and normal findings (*n* = 3, 10%). In 27 patients, the capsule was removed surgically and, in one patient, by means of double-balloon enteroscopy. Two of the patients still have the capsule retained after 2 years of expectation. One patient with CD and a stricture had the capsule retained for 2.5 years, and thereafter spontaneous passage occurred. During this time, the patient was treated with TNF-alfa-antibodies. 

Severe obstructive post-CE symptoms were reported from seven patients. Symptoms of obstruction appeared 0–4 weeks after the CE-examination. In six of the patients, intestinal obstruction with the capsule remaining in the affected area was confirmed by radiology, and in the seventh patient the capsule was found proximal to the obstruction. The surgical procedures were performed as acute or semiacute, 1-2 days after the radiology examination in six patients. One patient had a radiological confirmed capsule retention, and in this case surgery was performed acute without a new radiology confirmation when the obstructive symptoms appeared. In all seven patients obstructive intestinal disease was found during surgery, but in three of the patients no capsule was found. The clinical evaluation was that CE had contributed to onset of the acute obstructive symptoms in six of the seven patients. 

Postoperative complications were reported in 3 of the 27 operated patients and two of them died a few days after surgery. The first patient had widespread malignant disease and died of multiorgan failure at the intensive care unit. The second patient was a 53-year-old man in whom acute surgery was performed due to obstructive symptoms. In this case, CD and strictures were found, and a small bowel resection was performed. He seemed to recover well and was released from the hospital five days later. Six days after surgery he suddenly collapsed at home and was dead on arrival to the hospital. Postmortem showed rupture of the anastomosis.

### 3.4. Risk Factors for Capsule Retention

The risk of capsule retention (analysed for patients from Centers 1 and 2) was not correlated to gender (*P* = 0.19) or age (*P* = 0.14). The highest risk was found in patients with previously known CD with an odds ratio of 9.39 (3.32–26.54, *P* < 0.001) compared with OGIB. Suspected tumor as the indication for CE was also connected to a higher risk of capsule retention (Table 3).

### 3.5. Differences in Completeness between the Capsule Systems

At Center 1, using the PillCam capsule, 695 of 842 examinations due to OGIB were completed (82.5%). The mean age was 60 years. At Center 2, using the Olympus capsule, 144 of 195 examinations due to OGIB were completed (73.9%). The mean age was 59 years. The difference in completeness of examination was statistically significant (*P* = 0.005).

## 4. Discussion

Capsule endoscopy has been a routine, first-line method to evaluate the small bowel since it was initiated about 10 years ago. It has been validated in a large number of studies, and, even though other methods for reaching the distant part of the small intestine have been developed, it is still widely used, probably because it has high sensitivity [22] and user-friendly management. We believe there is a need to continuously validate a method, especially when it achieves widespread use. This large study evaluating all CE investigations over a six-year period in Stockholm County shows a low rate of retentions affecting, in particular, patients with known CD or suspected tumor. The overall prognosis and outcome of the patients with capsule retention due to a benign disease were good although one fatal case occurred as a result of surgical capsule retrieval.

The total number of CEs and complete data on patients with an incomplete CE were available from all hospitals, but complete data on all patients undergoing CE were only possible to obtain from Centers 1 and 2 (which performed 85% of all the CE examinations). It is a weakness of this study that these data from Centers 3 and 4 are missing when calculating the odds ratios of incomplete examination and capsule retention. However, it is unlikely that the missing data would have had a great impact due to the relatively low number of CEs performed at these centers. 

Slow gastric transit with the capsule harboured in the stomach for the entire recording time was found in 14% of incomplete examination cases and was obviously a factor lowering the success rate. Use of prokinetics has not been shown to improve gastric transit times [8, 10]. A more frequent use of a real time viewer with manual placement of the capsule in the duodenum, in the case of gastric retention, may improve the possibility of complete transit. 

Male gender and older age were identified as risk factors for incomplete examination, but these factors were not of statistical significance when considering capsule retention. Older age comes with higher risk of concomitant diseases like diabetes, and this could be an explanation for age as a risk factor. 

The two most common capsule systems were compared in terms of completed examinations. The PillCam capsule was superior to the Olympus capsule in this aspect. We could not find any obvious reason for this difference between the two capsule systems. 

Follow up of patients with capsule retention showed overall good recovery in patients with nonmalignant disease. In most patients, capsule endoscopy contributed to the diagnosis, and patients were relieved from symptoms due to tumors or strictures after surgery. However, one patient, a 53-year-old man with CD, died because of a rupture of the anastomosis six days after surgery. This is probably a rare case, since CE has been proven safe in numerous studies [7, 9, 13, 19] but reflects the risk of serious adverse events in a large series of patients. 

Acute or semiacute surgery was performed in seven cases due to obstructive symptoms in patients with capsule retention. Rather surprisingly, the capsule was not found during surgery in three of these patients. One theory is that the capsule caused symptoms while passing through the stricture. The capsule might also have returned to a more proximal position in the small intestine. The clinical conclusion was that CE had contributed to onset of acute obstructive symptoms in at least six of the seven patients although the underlying disease of course was the main reason for intestinal obstruction. Obstructive symptoms due to impaction of the capsule were reported in other studies but at a lower frequency [13, 15, 23]. This study indicates that it might be more common than previously thought. 

In this study, known CD was the indication of highest risk for capsule retention which is in accordance with previous literature [9, 17]. Suspected tumor was also shown to be a risk factor. In a large multicenter study, findings of tumors were shown to be associated with capsule retention in 9.8% [18]. In the case of a stricturing tumor, the patient usually needs surgery anyway, and the capsule then can be removed. A stricture in a patient with CD could have been asymptomatic and still prevented the passage of the capsule, [17] and thus, there is a risk of unnecessary surgery if the capsule has to be removed. On the other hand, CE can be a way of finding a significant stricture that needs surgical intervention [24]. The use of a patency capsule before CE is a method to lower the risk of capsule retention [21] but also excludes some patients from being diagnosed by CE. Advantages and disadvantages of CE examination should be considered carefully, in particular, when high risk patients are involved. 

The most common means of retrieval of the capsule in this study was surgical; however, in the future, device-assisted enteroscopy will probably be used increasingly as an alternative to surgery [23]. Our results also support this management.

## 5. Conclusion

Incomplete examination was found in 20% of all CE examinations and is a major limitation of the method. Capsule retention was found in 1.3% of the CE examinations. Older age, male gender, known and suspected CD, and suspected tumor are connected to a risk of incomplete CE examination. Known CD and suspected tumor are also risk factors for capsule retention. CE has a low complication rate, but it includes a small risk of obstructive symptoms and a need for surgical intervention in the case of capsule retention.

##  Authors' Contribution

C. M. Höög and U. Sjöqvist designed the study concept, analyzed data, and wrote the paper. O. Broström contributed important advice and assisted with revision of the paper. All authors contributed with study material and data analyzing.

##  Conflict of Interests

The authors declare that there is no conflict of interests.

## Figures and Tables

**Table 1:** Odds ratio of obtaining an incomplete CE for different indications (adjusted for age and gender).

Diagnose	Odds ratio	Significance	95% C.I
OGIB	1	—	—
Suspected CD	1.45	*P* = 0.018	1.06–1.96
Known CD	3.74	*P* < 0.001	2.52–5.58
Tumor	1.81	*P* = 0.006	1.19–2.76
Other	2.051	NS	1.15–3.66

**Table 2:** Patients with capsule retention.

Nr	Age/gender*	Indication for CE	Findings in CE	Acute obstruction	Findings in surgery	Outcome after one year
1	53/M	OGIB	CD	No	CD and stricture	Well**
2	28/M	OGIB	Ongoing bleeding	Yes	Lymphoma, capsule found but not responsible for obstruction	Died two months later (rupture of anastomosis)
3	77/F	OGIB	Normal	No	Lymphoma	Died two month later (widespread disease)
4	81/F	OGIB	Stricture	No	Stricture (radiation)	Regress of bleeding
5	75/M	Tumor	Normal	No	Adenocarcinoma	Well
6	59/M	OGIB	Normal	No	Stricture of anastomosis	Well
7	40/M	Other	Tumor	No	Metastasis of teratoma	Lost for followup
8	34/M	OGIB	Tumor	No	Adenocarcinoma	Dead (wide-spread disease)
9	43/F	CD?	CD	Yes	CD, stricture	Well
10	14/F	Known CD	CD and stricture	No	CD, stricture	Well
11	64/F	OGIB	Stricture	No	Stricture (radiation)	Well
12	78/F	Tumor	Intestinal dilatation	Yes	Adenocarcinoma—widespread, capsule found in the stomach	Died 4 days post.op (Multiorgan-failure)
13	73/F	Tumor	Erosions	No	Stricture	Lost for followup
14	18/M	Known CD	CD, stricture	No	CD, stricture	Well
15	53/M	Known CD	CD, stricture	Yes	CD, stricture, capsule not found	Died 6 days post.op (rupture of anastomosis)
16	32/M	Known CD	CD	Yes	CD, stricture	Well
17	23/M	Known CD	CD, stricture	No	Declined retrieval, capsule retained 2 years then passed out	Well
18	58/F	Known CD	CD, stricture	Yes	CD, stricture	Well
19	79/F	OGIB	Stricture	No	Stricture (radiation), capsule not found	Well
20	16/M	Known CD	CD	No	Declined retrieval before, op now planned	Well (2,5 years)
21	48/M	CD?	CD	No	No, declined retrieval	Well (2 years)
22	64/M	OGIB	CD, stricture	No	CD, stricture	Abdominal pain
23	67/F	Known CD	CD	Yes	CD, ileus (adhesions)	Well
24	49/F	CD?	Stricture	No	Stricture (radiation)	Well
25	53/M	OGIB	Tumor	No	Capsule retrieved by means of DBE***, PAD = adenocarcinoma	Well
26	33/F	Known CD	CD, stricture	No	CD, stricture	Well
27	67/M	Known CD	CD, tumor	No	Adenocarcinoma	Well
28	38/F	Known CD	CD	No	CD, stricture	Well
29	63/F	CD?	Erosions	Yes	Stricture (radiation), perforation, capsule not found	Well
30	48/M	Known CD	Erosions	Yes	CD, stricture, capsule not found	Well
31	50/M	Tumor	Tumor	No	Metastasis of teratoma	Well

*M: Male, F: Female, **Well: no clinical symptoms such as bleeding, abdominal pain, or diarrhea, ***Double balloon enteroscopy.

**Table 3:** Odds ratio of capsule retention of the capsule for different indications (adjusted for age and gender).

Diagnose	Odds ratio	Significance	95% C.I
OGIB	1	—	—
Supected CD	0.76	NS	0.18–3.08
Known CD	9.39	*P* < 0.001	3.32–26.54
Tumor	3.88	*P* = 0.026	1.18–12.81
Other	3.99	NS	0.80–19.87
